# Group A streptococcal strains isolated in Lao People's Democratic Republic from 2004 to 2013

**DOI:** 10.1017/S0950268815002927

**Published:** 2015-12-08

**Authors:** S. RATTANAVONG, D. A. B. DANCE, V. DAVONG, C. BAKER, H. FROST, R. PHETSOUVANH, M. VONGSOUVATH, P. N. NEWTON, A. C. STEER, P. R. SMEESTERS

**Affiliations:** 1 Lao-Oxford-Mahosot Hospital Wellcome Trust Research Unit, Microbiology Laboratory, Mahosot Hospital, Vientiane, Lao People's Democratic Republic; 2 Centre for Tropical Medicine and Global Health, Old Road Campus, University of Oxford, Oxford, UK; 3 Group A Streptococcus Research Group, Murdoch Children's Research Institute, Melbourne, Australia; 4 Centre for International Child Health, University of Melbourne, Melbourne, Australia; 5 Department of Paediatrics, Hôpital Universitaire des Enfants Reine Fabiola, Brussels, Belgium

**Keywords:** Molecular epidemiology, *Streptococcus pyogenes*, vaccines

## Abstract

Epidemiological data regarding group A streptococcal (GAS) infections in South East Asia are scarce with no information from Laos. We characterized *emm* types, *emm* clusters and the antibiotic resistance profile of 124 GAS isolates recovered in Laos during 2004–2013. Most strains were recovered from skin and invasive infections (76% and 19%, respectively). Thirty-four *emm* types were identified as belonging to 12 *emm* clusters and no novel *emm* types were identified. No significant differences were observed in the distribution of *emm* types or *emm* clusters according to age or site of recovery (skin or invasive infections). There was moderate strain diversity in this country but considerable differences in *emm*-type distribution between Laos, Thailand and Cambodia. Vaccine coverage was high for the J8 vaccine candidate. The theoretical coverage for the 30-valent vaccine candidate needs further investigation. Antibiotic resistance was moderate to erythromycin and chloramphenicol (8% and 7%, respectively) and low to ofloxacin (<1%).

Group A streptococci (*Streptococcus pyogenes*; GAS) cause significant morbidity and mortality globally with most of the disease burden occurring in low- and middle-income settings. With no effective control strategies available, a GAS vaccine is urgently needed. The most advanced vaccine candidates use the surface M protein as antigen. Two vaccine candidates have entered phase 1 clinical trials over the past decade with a further two vaccines planned for trials in 2015 [1]. The 30-valent type-specific vaccine candidate includes peptides from a selection of M proteins associated with disease burden in both high- and low-income settings but questions have been raised regarding coverage in low-income settings where a high diversity of circulating *emm* types has been observed [2, 3]. The J8 vaccine is based on a conserved region of the M protein (J8) and aims to provide broad protection across many strains. However, no fewer than 68 allelic variants have been described for J8 and the relationship between allelic diversity and vaccine efficacy has not yet been systematically characterized [1]. Importantly, limited epidemiological data regarding circulating *emm* types and J8 variants are available from a number of key regions of the world making vaccine-coverage estimates imprecise, especially from South East Asia with only one study originating from a low-income country (Cambodia) [2–4].

The Lao People's Democratic Republic (Laos) is a low- to middle-income country with a population of ~6·9 million people which, despite rapid economic growth, is still one of the poorest in South East Asia (http://data.worldbank.org/country/lao-pdr). It also has some of the worst health indicators in the region, with an average life expectancy of 65 years for males and 68 years for females, and an infant mortality rate of 54/1000 live births in 2013 (http://apps.who.int/gho/data/view.main.CM1320R?lang=en). In addition, very few diagnostic laboratories undertake bacterial culture, therefore data on the epidemiology and antimicrobial susceptibility of bacterial pathogens within Laos are very scarce.

The Microbiology Laboratory of Mahosot Hospital, the largest hospital in the capital, Vientiane, has conducted culture-based diagnosis of bacterial infections and storage of significant pathogens since 2000. We analysed these databases to identify GAS isolates recovered during a 10-year period (2004–2013). We extracted clinical details and demographic information of patients with GAS infection, including geographical coordinates of the addresses of the patients. Invasive disease was defined as the isolation of GAS from blood in a patient with a clinical infection. Skin infection, acute otitis media, and pharyngitis was defined by the presence of clinical symptoms associated with the isolation of GAS from the relevant site.

Identification of GAS was based on colony morphology, *β*-haemolysis on 5% goat blood agar, negative catalase reaction, and detection of Lancefield Group A antigen by latex agglutination (Streptococcal Grouping kit, Oxoid, UK). Antibiotic susceptibility to four antibiotics (penicillin, erythromycin, ofloxacin, chloramphenicol) was determined by the CLSI disk diffusion method and isolates were frozen at −80 °C prior to shipment to Melbourne for further testing. The isolates were re-confirmed as GAS as above and were then *emm*-typed according to the US Centers for Disease Control and Prevention protocol with minor modifications; primers MF2 and MR1 were used when primers 1 and 2 were not successful, as described previously [5]. In addition to *emm*-typing, we also classified isolates into *emm* clusters. This typing system classifies the many GAS *emm* types into 48 discrete *emm* clusters containing closely related M proteins that share binding and structural properties [6–8]. *emm* clusters can be directly deduced by the *emm*-typing result and predict the J8 vaccine antigen content [6]. Coverage by the 30-valent vaccine was estimated using the latest cross-opsonization data [6, 9, 10]. We used Simpson's Reciprocal Index (SRI) to assess strain diversity [3]. Regional molecular epidemiology was assessed by comparison of the data with a similar dataset from Thailand [11] and Cambodia [4].

The Oxford Tropical Research Ethics Committee and National Ethics Committee for Health Research, Government of Laos approved this study.

We characterized 124 GAS isolates. Median patient age was 35 years (interquartile range 7–56, range 0–92; data available for 121/124 patients) and 31·4% of the patients were aged <15 years at presentation. Most of the patients were diagnosed with a skin infection (94/124, 76%) followed by invasive infection (24/124, 19%). There was no clinical information available for four patients. We identified 34 *emm* types as belonging to 12 *emm* clusters and no novel *emm* types were identified (Table 1). No significant differences were observed in the distribution of *emm* types or *emm* clusters according to age or site of recovery (skin or invasive infections). Four isolates were non-typable. While there were 34 different *emm* types observed among 124 GAS isolates in this study, the strain diversity as measured by the SRI (that takes into account both richness and evenness), was 13·2 [95% confidence interval (CI) 9·6–21·5]. This indicates a relatively moderate strain diversity in comparison to data from children in Cambodia (SRI = 28·5) and other other low-income countries [2, 4]. Geographical coordinates of the patient's home were available for 117/124 patients included in this study and showed that 96% (112/117) of patients' homes were within ~100 km radius of Vientiane, the main catchment area of Mahosot Hospital. This relatively small geographical area may partially account for the relatively low diversity of strains observed.

**Table 1.** *emm* clusters and *emm* types of the GAS isolates characterized in the study

*emm* cluster	Isolates (*n*)	*emm* types (*n*)
E3	47	25 (6), 44 (28), 49 (2), 58 (4), 79 (3), 82 (4)
E4	21	8 (1), 22 (5), 73 (2), 77 (2), 89 (1), 102 (1), 109 (8), 114 (1)
E2	15	76 (4), 104 (5), 106 (4), 110 (2)
E6	12	11 (1), 63 (2), 75 (6), 81 (3)
D4	8	33 (2), 70 (1), 101 (1), 230 (4)
M95	4	95 (4)
Non-typable	4	Non-typable (4)
E1	3	4 (1), 165 (2)
M74	3	74 (3)
M105	3	105 (3)
M185	2	185 (2)
A-C3	1	1 (1)
D2	1	100 (1)

No isolate was resistant to penicillin; however, one isolate was resistant to ofloxacin (another was intermediate), nine (7%) were resistant to chloramphenicol (three intermediate) and 10 (8%) were resistant to erythromycin. These chloramphenicol- and erythromycin-resistant isolates belonged to seven and six different *emm* types, respectively. Of note, seven of the 10 erythromycin-resistant isolates were also resistant to chloramphenicol. These data represent the first information about GAS antibiotic resistance in South East Asia. Data from East Asia indicate rates of erythromycin resistance ranging from 8% to 14·6% in Taiwan during the period 2010–2011 [12, 13], reaching as high as >95% in China [14].

Sixty-one (49%) isolates belonged to *emm* types included in the 30-valent vaccine candidate. Thirty-eight (31%) isolates were *emm* types that have been shown to be cross-opsonized in a rabbit model and therefore have the potential to be covered by the 30-valent vaccine. Taking into account this cross-opsonization effect, the theoretical coverage could be 80% (95% CI 73–87), but may be even higher since 19% of the isolates belonged to eight *emm* types that had not yet been tested for cross-opsonization. These eight *emm* types should be investigated for potential cross-opsonization in order to better assess the theoretical coverage of the 30-valent vaccine in Laos. In terms of theoretical coverage of the J8 vaccine candidate, 19 (15%) and 101 (82%) of the isolates were predicted to contain the J8 and J8·1 allele, respectively. Therefore, J8 vaccine coverage could be expected to be 97% (95% CI 94–100).

We compared these data to previous data from Thailand and Cambodia. The Thailand dataset comprises 106 GAS isolates from skin and throat collected between 1985 and 2004, including those from patients with rheumatic heart disease [11]. The Cambodian dataset comprises 150 GAS isolates recovered from skin, throat and invasive infections in children collected between 2007 and 2012 [4]. The *emm*-type distribution between these three countries shows that nearly half of the Cambodian isolates belong to an *emm* type which has not been recovered in the two other countries (Fig. 1). By contrast, only 10 and 12% of the strains isolated in Thailand and Laos, respectively, belonged to an *emm* type not recovered in the other two countries. The *emm* cluster distribution shows that 86–97% of the strains belong to an *emm* cluster present in all three countries (Fig. 1). Moreover, the distribution also shows that the apparently distinct epidemiology of GAS in Cambodia does not correlate with the presence of specific *emm* clusters.

**Fig. 1.**
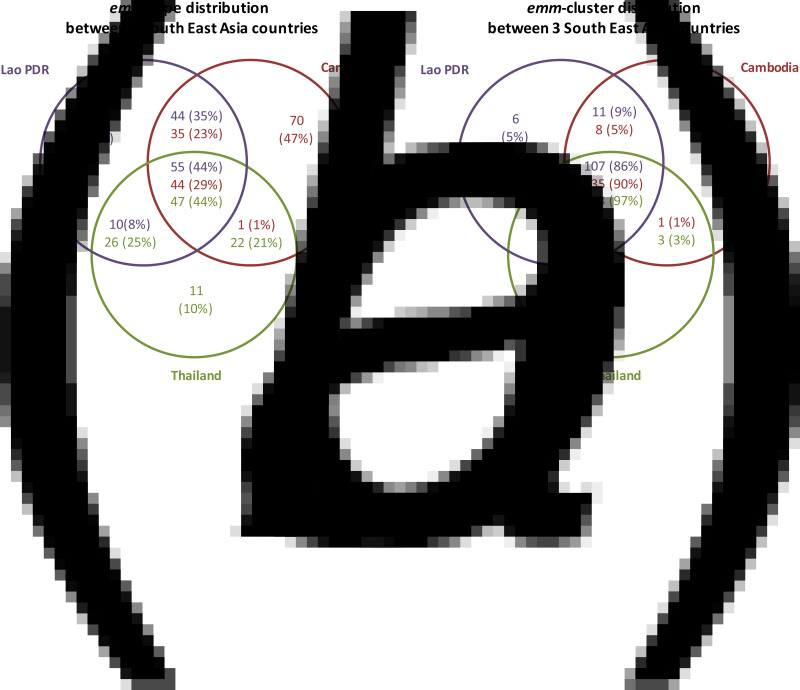
Comparison of the GAS isolates recovered from 3 South East Asian countries. Distribution of *emm* types and *emm* clusters among GAS infections recovered from three Southeast Asian countries. The number of isolates (%) associated with infections and belonging to specific and common (*a*) *emm* types and (*b*) *emm* clusters is shown for each country.

The most important limitation of our study is its retrospective nature. In future, prospective and comprehensive GAS infection surveillance in Laos will be highly desirable. Other limitations of our study are the relative absence of pharyngeal isolates from the collection and the restricted geographical sampling area. Finally, caution should be exercised when comparing the Lao, Thai and Cambodian datasets given the methodological differences in sampling and study periods. Notably, a potential confounding factor for the differences in strain distribution is that the Cambodian isolates were all from children. Better prospective data obtained using similar methodologies in terms of study period, inclusion criteria and targeted population should be obtained before firm conclusions can be drawn.

Overall, these data indicate a relatively low diversity of circulating GAS strains in the central region from Laos, a high coverage of the J8 vaccine candidate and the need for complementary studies to assess the potential coverage of the 30-valent vaccine candidate. These results also confirm the importance of epidemiological studies at a local and regional level to inform vaccine development.
